# Longitudinal relation between state-trait maternal irritability and harsh parenting

**DOI:** 10.1371/journal.pone.0209493

**Published:** 2019-01-09

**Authors:** Eriona Thartori, Antonio Zuffianò, Concetta Pastorelli, Maria Gerbino, Carolina Lunetti, Ainzara Favini, Emanuele Basili, Laura Di Giunta, Dario Bacchini, Jennifer E. Lansford

**Affiliations:** 1 Department of Psychology, Sapienza University of Rome, Rome, Italy; 2 Department of Psychology, Liverpool Hope University, Liverpool, United Kingdom; 3 Department of Humanities, Università degli Studi di Napoli Federico II, Naples, Italy; 4 Center for Child and Family Policy, Duke University, Durham, North Carolina, United States of America; Icahn School of Medicine at Mount Sinai Hospital, UNITED STATES

## Abstract

According to Belsky’s process model of parenting, parents’ personality represents the most important factor influencing parenting and child development. While an extensive literature has empirically corroborated the role of irritability traits in predicting aggressive behaviors in laboratory-based studies, only a few studies have examined the role of irritability in predicting aggressive behaviors within family contexts. The present study addressed this gap by examining the longitudinal association between maternal irritability and harsh parenting. Referencing latent state-trait theory (LST), first we estimated the amount of variance in mothers’ irritability due to trait and state components, and, next, we examined the relation between mothers’ irritability (both at trait- and state- levels) and harsh parenting over time. A sample of 204 mothers from Naples and Rome provided data over 5 years in four waves. Mothers averaged 40.30 years (*SD* = 5.33) at Time 1 and 44.01 years (*SD* = 5.43) at Time 4. Their children (50% girls) were 9.45 years (*SD* = 0.74) at Time 1 and 13.18 years (SD = 0.66) at Time 4. Results of LST analysis showed that, on average, 39% of variability in irritability was due to trait-like factors and only 12% to state-like factors. A multitrait-multistate model revealed that the irritability trait associated with mother’s lack of control predicted her harsh parenting 1-year later, while controlling for the rank-order stability of harsh parenting.

## Introduction

Developmental and clinical studies have empirically supported the detrimental consequences of harsh parenting (both physical and verbal) for children’s emotional and behavioral health. For instance, in a meta-analysis of 88 studies conducted over the last 62 years [1], parental physical punishment was associated with increased children’s aggression, delinquency, antisocial behavior, and diminished moral internalization, poorer quality of parent-child relationships, and lower mental health. Similarly, the use of harsh verbal parenting (e.g., shouting, yelling, threatening) has been associated with children’s mental problems such as depressive symptoms, low self-esteem, and problematic social interaction (e.g., [2–5]). Yet, despite the negative effects on children development, the use of harsh parenting practices is still present across several societies and cultures [6]. A systematic review [7] using population-based data from 112 studies in 96 countries revealed that over 1 billion children and adolescents (from 2 to 17 years of age) have experienced physical, emotional, and/or sexual violence in the previous past year. Focusing on the use of physical discipline against children, like spanking or slapping, cross-cultural findings from 336 mother-child dyads from China, India, Italy, Kenya, Philippines and Thailand showed that the rate of endorsement and use of corporal punishment was moderately high across cultures [8]. Specifically, the rank order (from low to high) of how often mothers reported using physical discipline was Thailand, China, the Philippines, Italy, India and Kenya [8].

Hence, it is important to identify the psychological determinants of harsh parenting to reduce the risk of escalation in parent-child aggression. Previous research focused on serious parental psychopathologies (e.g., depression; e.g., [9]) as determinants of harsh parenting practices, thereby partially neglecting the role of parental personality characteristics in affecting their parenting behavior. In the present study, we aimed to address this research gap by investigating the longitudinal association between mothers’ irritability and harsh parenting over 5 years. Specifically, using Latent State-Trait modeling, we tried to clarify whether mothers’ harsh parenting was predicted by their irritability at the trait-level (i.e., their general disposition to be irritable) versus state-level (i.e., being more irritable than usual at a specific occasion).

### Maternal irritability and harsh parenting

The Irritability trait has been defined by Caprara et al. [10] as the enduring tendency to react impulsively, aggressively, and rudely at the slightest provocation and disagreement. It is a personality dimension related to individuals’ capacities to tolerate frustration as well as to dominate their reactions in either real or apparent situations of danger, offense, or attack [10]. Previous experimental studies investigating the frustration-aggression hypothesis showed that participants with high levels of irritability engaged in higher levels of aggressive behaviors (i.e. delivering noxious stimulation against an innocent peer) under both neutral and provoking conditions (e.g. for a review see [11]) compared to those with low levels of irritability. However, while an extensive literature has empirically corroborated the role of irritability trait on aggressive behaviors in experimental studies, only a few studies [12, 13], conducted in North American settings, investigated the positive association between irritability trait and aggressive behaviors within more naturalistic family contexts. For example, in a study with 206 families with children attending fourth grade, Greenwald et al. [12] found that parental irritability coupled with ineffective parenting led to escalated punitive practices toward the child (e.g., harsh physical punishment). In another study with 122 mothers and their children aged from 4 to 8 years, Shay and Knutson [13] found that maternal irritability mediated the existing relation between maternal depression and harsh parenting. In other words, they showed that maternal irritability contributed to the escalation of discipline in response to child transgressions by a depressed mother. Consistent with coercion theory [14], previous cross-sectional family studies have found that mothers’ dispositional tendency to be irritable and to react aggressively at the slightest provocation set the stage for coercive discipline, increasing the risk for problem child behavior (for a review see [1]), which in turn increase the risk for harsher and more punitive parenting [12].

Because of the cross-sectional nature of the aforementioned family studies and their focus on the childhood period, in the present study we sought to clarify whether mothers’ irritability trait longitudinally predicts their harsh parenting during their children’s transition to adolescence. This is particularly relevant because several studies have shown that the persistence of harsh parenting across different time periods has a stronger detrimental effect on children’s negative outcomes (e.g., [15–18]). Moreover, because contextual factors, such as daily hassles related to family life or to child misbehavior, may further exacerbate one’s own general irritability level (trait-level), we were interested to examine whether the mothers’ momentary, occasion-specific deviations from their irritability trait level (due to mothers’ situational circumstances when the measurements were performed; i.e., state-level) could influence their harsh parenting.

### Conceptual framework: Latent State-Trait theory

In the present study we used Latent State-Trait analysis (LST) to estimate the impact that both the maternal disposition to react irritably and the momentary deviations from her general trait due to situational circumstances have on predicting later harsh parenting. This is theoretically relevant because, even if previous cross-sectional studies (e.g., [12, 13]) have shown that the tendency to be irritable is associated with harsh parenting, we cannot exclude that the strength of this correlation could be affected by the fact that in specific moments the parents were more irritable than usual due to situational circumstances, and this momentary deviation from their general level of irritability (trait-like) could explain their tendency to behave in an aggressive way toward children. Therefore, to verify the robustness of the association between parental irritability trait and harsh parenting we should disentangle the effect that is attributed to the situational circumstances (i.e., state-like) that make parents more prone to be irritable and to act in an aggressive way from the effect that is attributed to their tendency to react aggressively and impulsively at the slightest provocation or disagreement (i.e., trait-like). LST analysis makes it possible to separate person effects (i.e., trait-like) from situation and person x situation interaction effects as well as measurement error in longitudinal studies in which a construct is assessed with multiple indicators [19–21]. The natural variation of situations between occasions and the difference between subjects (persons-in-situations) within each occasion of measurement is sufficient to separate trait from situational effects [19–21].

## The present study

Following Belsky’s [22] process model of parenting that assigns a primary importance to parent personality in predicting parenting, and, consequently, child development, the present study extended previous research by examining the longitudinal relation between maternal irritability and harsh parenting during the transition to adolescence. Specifically, the aim of the present study was twofold. First, referencing LST [19–21], we disentangled at item-level the variance of mothers’ irritability due to dispositional tendencies (i.e., trait-like) and situational circumstances (i.e., state-like) to understand to what extent maternal irritability could be attributed to the mother’s stable tendency to react irritably or the time-specific situational effects. Based on the scale construction and related validation studies [10], we expected that irritability reflects more trait- than state- components. Second, we examined the effects of maternal irritability at both trait- and state-levels in predicting mothers’ harsh parenting one year later. Although previous cross-sectional studies showed the positive relation between maternal irritability at the trait-level and harsh parenting [12, 13], to the best of our knowledge, our study represents a first attempt to clarify the contribution played by both trait and state components in a longitudinal framework. We expected that mothers’ irritability trait as well as their positive deviations from their own general level of irritability could predicted their harsh parenting over time.

## Method

### Participants

A convenience sample of 204 mothers from Rome (*n* = 105) and Naples (*n* = 99) provided data over five years across four waves. Families were recruited from schools that serve socioeconomically diverse populations. Children’s (50% girls) mean age was 9.45 (*SD* = 0.74) at Time 1 (T1) and 13.18 (SD = 0.66) at Time 4 (T4). Mothers’ mean age was 40.30 years (*SD* = 5.33) at T1 and 44.01 years (*SD* = 5.43) in T4. Mothers had completed 11.86 (*SD* = 4.49) years of education on average. At T1, 83.8% of mothers were married, 1.5% divorced, 6.2% separated, 1% widowed, 3% cohabiting, 1.5% remarried, and 3% never married. Nearly all were biological mothers (98%), with 2% being grandmother, stepmother or another mother figure.

### Attrition and missing data analyses

The participation rate was high during the longitudinal data collection: 93% from T1 to T4. The attrition was mainly due to the unavailability of individuals to take part in the later phases of the study or our inability to contact the participant. Importantly, our data also met the strict assumption of missing completely at random (MCAR) as the Little’s Test [23] was not statistically significant, χ^2^(126) = 137.126, *p* = 0.708, suggesting the missingness on one variable is unrelated to the other measured or unmeasured variables. Accordingly, full information maximum-likelihood (FIML) in M*plus* 7.0 [24] was used to handle missing data [25], enabling us to include all available data in the analyses. FIML does not estimate the missing data, rather it fits the covariance structure model directly to the observed and available raw data for each participant, offering unbiased estimates under the assumption that the missing data are missing at random [25].

### Procedure

Institutional review board at Sapienza University of Rome approved the study protocol. After obtaining parental informed consent and child assent, interviews were conducted in participants’ homes or locations of their choosing (e.g., at the university) by expert interviewers who were trained by the investigator responsible for data collection and the investigator’s staff by using a structured training protocol. Interviews lasted approximately 1.5–2 hr. Parents were given modest financial compensation for their participation.

### Measures

#### Irritability

Mothers rated their irritability using a 4-item version of the Irritability Scale [10]. The items were “When I am tired I easily lose control,” “When I am irritated I can’t tolerate discussions,” “I often feel like a powder keg ready to explode,” and “Some people irritate me if they just open their mouth.” Each item was scored on a 6-point scale ranging from 1 (*completely false for me*) to 6 (*completely true for me*). Items were averaged to create an irritability scale in each year. Omega coefficients were .68 (T1), .63 (T2), .73 (T3), and .73 (T4).

#### Harsh parenting

Mothers rated their harsh parenting using items developed by UNICEF [26] for their Multiple Indicator Cluster Survey. The items were selected by convening an international panel of 25 experts to identify candidate items from existing validated measures of caregiving. The items that resulted from this process were adapted from the Parent-Child Conflict Tactics Scale [27] and the WorldSAFE survey questionnaire [28]. Mothers were asked whether they or anyone in their household had used each of seven forms of harsh parenting (shook the child; spanked, hit, or slapped the child with a bare hand; hit the child with a belt or other hard object; hit or slapped the child on the hand, arm, or leg; hit or slapped the child on the face, head, or ears; shouted, yelled, or screamed the child; called the child dumb, lazy, or another name like that) with the target child in the last month (1 = *no*, 2 = *yes*). Items were averaged to create a harsh parenting scale in each year. Omega coefficients were .70 (T1), .60 (T2), .69 (T3), and .75 (T4).

### Analytical approach

Preliminarily, means, standard deviations, reliability and zero-order correlations among the study variables were calculated using SPSS (Version 23, SPSS Inc., Chicago, IL). Then, we used LST models [21, 29, 30] to disentangle the proportion of irritability variance due to trait and state components, and measurement error. The decomposition of the variance at item-level was performed using the four time points. Finally, we tested maternal irritability trait and state components as predictors of harsh parenting over time using an LST model. To properly establish a temporal order between mothers’ irritability trait components (the predictors) and their harsh parenting (the outcome), mothers’ irritability was modeled using only the first three time points (i.e., when children were 9, 10, and 12 years, respectively). This allowed us to test the role of trait irritability as a predictor of 1-year later harsh parenting, when children were 13 years old (see S3 Fig).

Evaluation of the goodness of fit for each model was based on standard procedures: χ^2^ likelihood ratio statistic, comparative fit index (CFI), Tucker-Lewis Index (TLI), and root mean square error of approximation (RMSEA) with 90% confidence interval (CI). Because the *χ2* is sensitive to large sample sizes, we accepted CFI and TLI ≥ .90, and RMSEA < .08 as indicative of acceptable model fit [31].

## Results

### Descriptive statistics

Means, standard deviations and zero-order correlations among the study variables are presented in Table 1.

**Table 1:** Descriptive statistics and correlations among study variables.

Variables	Mean	*SD*	1	2	3	4	5	6	7	8
1. Irritability T1	3.61	1.11	1							
2. Irritability T2	3.59	1.10	.58***	1						
3. Irritability T3	3.47	1.16	.53***	.53***	1					
4. Irritability T4	3.47	1.14	.56***	.51***	.49***	1				
5. Harsh parenting T1	1.33	.23	.26***	.13	.16*	.18*	1			
6. Harsh parenting T2	1.30	.21	.25***	.20**	.21**	.27***	.51***	1		
7. Harsh parenting T3	1.27	.20	.19**	.08	.20**	.19*	.48***	.49***	1	
8. Harsh parenting T4	1.27	.22	.15*	.06	.11	.28***	.38***	.49***	.47***	1

*Note*. T = time. *SD* = Standard deviations.

* *p* < .05.

** *p* < .01.

*** *p* < .001.

### LST models

We performed LST analysis that allows decomposing the variance of an observed variable into variance that is due to the trait, the state, and the measurement error. First, we tested a singletrait-multistate model (STMS; see S1 Fig) in which (a) all indicators measured a single common latent trait factor, ξ and (b) all indicators assessed at the same time point *t* measured a common latent state residual factor ζ_*t*_, [21, 30]. The STMS model presented a poor fit, χ^2^(121) = 300.016, *p* < .001, CFI = .80, TLI = .81, RMSEA = .085 (90% CI = .073–.097). Sequential fit diagnostic evaluation analysis (i.e., modification indices) indicated a misfit due to error covariance between the same indicator *i* over time, suggesting that indicator-specific trait factors should be estimated to obtain a more realistic model [21, 29, 30]. Then, a multitrait-multistate model (MTMS; see S2 Fig) was tested in which there were (a) 4 trait-specific indicators, ξ_*i*_ and (b) all indicators assessed at the same time point *t* measured a common latent state residual factor ζ_*t*_ [29, 30]. Compared to the STMS model, the MTMS showed a better fit χ^2^(115) = 152.467, *p* < .05, CFI = .96, TLI = .96, RMSEA = .04 (90% CI = .020–.056). As shown in Table 2, squared standardized loadings indicated that approximately 32–45% of the variance of the items reflected trait-level variability whereas only 7–21% reflected state-level variability. As shown in Table 3, although on average a large part of variability of the items was unexplained by the MTMS model, mothers’ irritability was mostly captured by trait-level tendencies rather than occasion-specific state irritability.

**Table 2:** Factor loadings, intercepts, and variances from MTMS model.

	Indicator	λ	γ	α	ɛ
T1	1. When I am tired I easily lose control.	1.000 (0.668)	1.000 (0.243)	0.000	1.033
	2. When I am irritated I can’t tolerate discussions.	1.000 (0.572)	1.213 (0.294)	0.000	1.230
	3. I often feel like a powder keg ready to explode.	1.000 (0.708)	2.134 (0.449)	0.000	0.824
	4. Some people irritate me if they just open their mouth.	1.000 (0.571)	1.619 (0.350)	0.000	1.450
T2	1. When I am tired I easily lose control.	1.000 (0.666)	1.000 (0.202)	0.000	1.082
	2. When I am irritated I can’t tolerate discussions.	1.000 (0.553)	1.213 (0.237)	0.000	1.430
	3. I often feel like a powder keg ready to explode.	1.000 (0.655)	2.134 (0.347)	0.000	1.461
	4. Some people irritate me if they just open their mouth.	1.000 (0.575)	1.619 (0.295)	0.000	1.511
T3	1. When I am tired I easily lose control.	1.000 (0.677)	1.000 (0.307)	0.000	0.911
	2. When I am irritated I can’t tolerate discussions.	1.000 (0.540)	1.213 (0.346)	0.000	1.379
	3. I often feel like a powder keg ready to explode.	1.000 (0.689)	2.134 (0.545)	0.000	0.667
	4. Some people irritate me if they just open their mouth.	1.000 (0.547)	1.619 (0.418)	0.000	1.510
T4	1. When I am tired I easily lose control.	1.000 (0.676)	1.000 (0.270)	0.000	0.958
	2. When I am irritated I can’t tolerate discussions.	1.000 (0.604)	1.213 (0.341)	0.000	0.972
	3. I often feel like a powder keg ready to explode.	1.000 (0.666)	2.134 (0.465)	0.000	1.066
	4. Some people irritate me if they just open their mouth.	1.000 (0.563)	1.619 (0.379)	0.000	1.465
	**Variances**				
	Trait variability (ξ1)	0.932	*p* < 0.001		
	Trait variability (ξ2)	0.685	*p* < 0.001		
	Trait variability (ξ3)	1.393	*p* < 0.001		
	Trait variability (ξ4)	0.859	*p* < 0.001		
	State variability (ζ1)	0.123	*p* = 0.005		
	State variability (ζ2)	0.086	*p* = 0.041		
	State variability (ζ3)	0.191	*p* = 0.005		
	State variability (ζ4)	0.149	*p* = 0.015		

*Note*. Item intercepts (α), residual variances (ɛ), unstandardized factor loadings, and standardized factor loadings (in parentheses) for irritability at both trait level (λ) and state level (γ) are reported. All factor loadings (λ and γ) were statistically significant at p < 0.001. MTMS = multi-trait multi-state model; T1 = Time 1; T2 = Time 2; T3 = Time 3; T4 = Time 4.

**Table 3:** Average consistency, occasion-specific, and error measurement estimates for Latent State-Trait analyses.

Indicator	Trait Consistency Con(τ_*it*_)	Occasion-Specificity Spe(τ_*it*_)	Error measurement (ɛ_*it*_)
1. When I am tired I easily lose control.	.45 (.44 –.46)	.07 (.04 –.09)	.48 (.45 –.51)
2. When I am irritated I can’t tolerate discussions.	.32 (.29 –.36)	.10 (.06 –.12)	.58 (.52 –.64)
3. I often feel like a powder keg ready to explode.	.46 (.43 –.50)	.21 (.12 –.30)	.33 (.23 –.45)
4. Some people irritate me if they just open their mouth.	.32 (.30 –.33)	.13 (.09 –.17)	.55 (.53 –.58)

*Note*. Entries indicate averages of the indicators across time (with ranges in parentheses).

### LST model relating mothers’ irritability and harsh parenting

**Fig 1:**
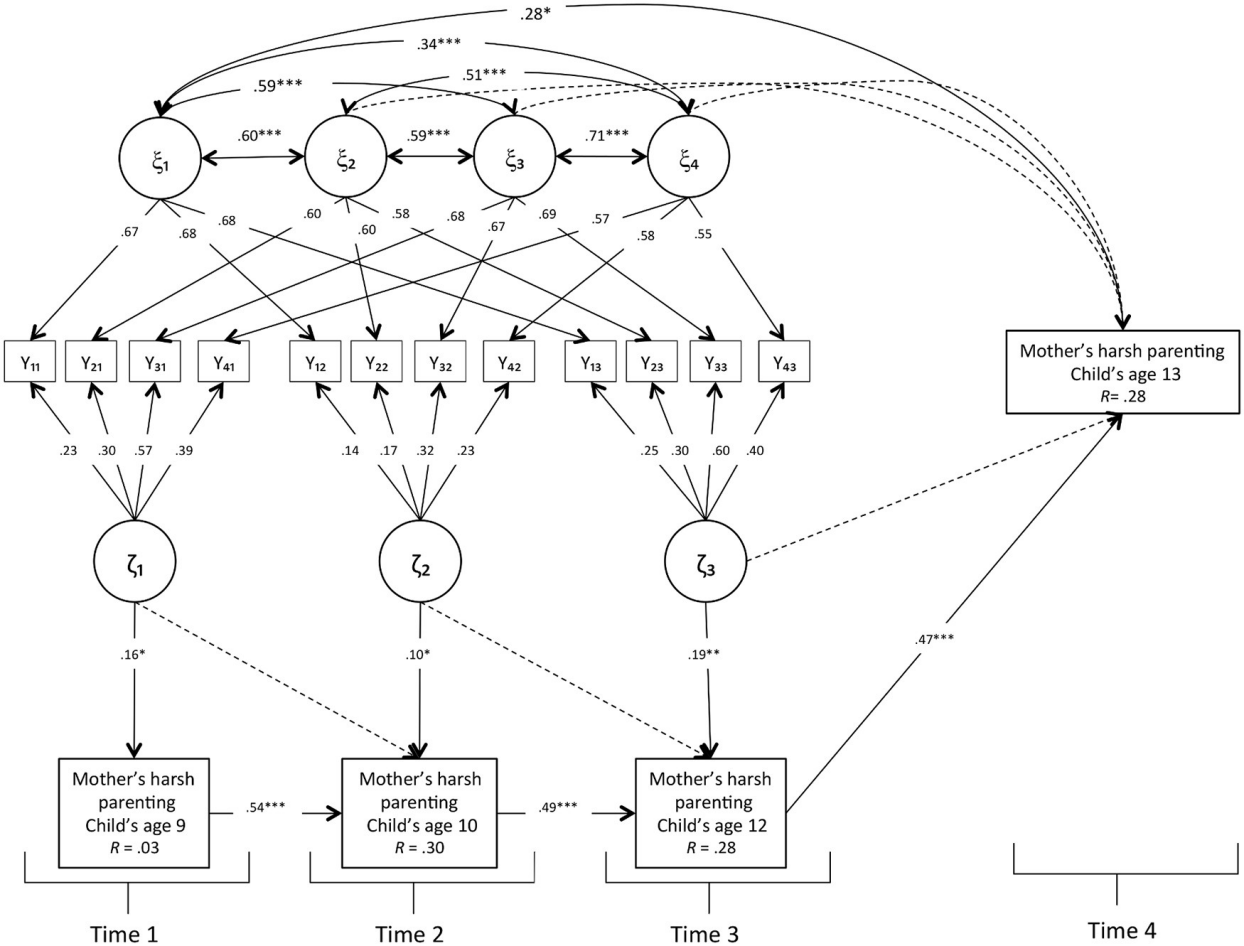
Results LST model relating mothers’ irritability and harsh parenting.

To examine the impact that both mothers’ irritability trait- (i.e., individual disposition) and state- (which reflected momentary deviations from the trait level at time *t*) components have on their harsh parenting, we performed a LST model which extends the previous MTMS model by including mothers’ harsh parenting as outcome variables. In particular, in order to test the predictive role of irritability trait components on later harsh parenting, mothers’ irritability was modeled using only the first three time points (i.e., when children were 9, 10, and 12 years, respectively; see S3 Fig). The LST model relating mothers’ irritability to their harsh parenting fit the data well, χ2(105) = 177.263, *p* < .001, CFI = .91, TLI = .90, RMSEA = .058 (90% CI = .043–.073). As shown in Fig 1, the irritability state factors predicted mothers’ harsh parenting concurrently, at each time point, but not across time. Conversely, indicator-specific irritability trait 1, which referred to the item “When I am tired I easily lose control,” predicted mothers’ harsh parenting one year later, when children were 13 years old, while controlling for the rank-order stability of harsh parenting. The model explained 28% of variance for mothers’ harsh parenting when children were age 13 years.

## Discussion

The present study represents a step forward in the study of irritability and aggressive behaviors in family contexts, for two reasons. First, it is the first study that aimed to disentangle the variance of irritability due to trait (i.e., mother stable disposition) and state (i.e., mothers’ situational circumstances when the measurement was performed) components, in order to clarify if the Irritability Scale developed by Caprara et al. [10], largely used in studies aimed to detect the personality trait predictive of aggressive behaviors (for a review see [11]), captured more stable trait or situation-specific effects. Second, it represents the first study that examined longitudinally the relation between mothers’ irritability, both at trait- and state- level, and their harsh parenting during their children’s transition to adolescence.

Concerning to the first aim of the present study, our findings suggest that Caprara and colleagues’ [10] scale measures stable characteristics in terms of individuals’ “tendency to react impulsively and aggressively to the slightest provocation or disagreement” (p. 3), showing that the irritability scale captured more trait variability (range from 29–50%) rather than situational-specific state variability (range from 4–30%). Although we are not aware of previous studies that specifically provided indications for interpreting the size of trait consistencies, we believe that these coefficients could be interpreted as large effects if we use Cohen’s cut-off for r-squared (i.e., *R*^2^ above 26% should indicate large effects). In addition, from a theoretical perspective, our trait-consistency coefficients were not highly different from those obtained in previous studies investigating the stability of similar personality characteristics (see for example [32]).

Our results supported empirically Deinzer and colleagues’ assumption [33] that any psychological questionnaires, even those that in theory are conceived as measures of stable traits, assess both individual dispositions and situational circumstances when the measurement is performed.

Regarding the longitudinal relation between mothers’ irritability (at both trait- and state- level) and harsh parenting, as shown in Fig 1, irritability state components predicted mothers’ harsh parenting concurrently, at each wave, but not across time, suggesting that mothers’ positive deviations from their irritability trait level (i.e., feeling more irritable than their own usual, average level) were associated with higher use of physical and verbal aggression toward the child. These irritability state variables did not predict mothers’ harsh parenting across time. Conversely, the indicator-specific trait irritability, which referred to the item “When I am tired I easily lose control,” predicted mothers’ harsh parenting 1 year later when children were 13 years old, while controlling for the rank-order stability of harsh parenting. This result provided empirical evidence that mothers’ irritability trait could longitudinally predict harsh parenting, characterized by the use of physical and verbal aggression against children [12, 13]. However, we found that only one latent trait indicator of maternal irritability predicted later harsh parenting. The predictive role of this indicator-specific latent trait factor on later harsh parenting, compared with the other irritability indicators, is interesting for two reasons. First, it represents one of the factors with the higher trait consistency (see Table 3). Therefore, we speculate that because this indicator captured more of mothers’ stable dispositional tendency to react aggressively and impulsively to the slightest provocation and disagreement rather than the situational circumstances that made the mothers irritable, it represented the stronger irritability indicator, which was able to predict mothers’ later harsh parenting once the situational effects were taken into account. Second, this indicator reflects a facet of irritability more anchored to impulsive reaction, which contributes to the prediction of mothers’ aggressive behavior toward the child.

## Limitations and future directions

Future research should address several limitations of the present study. First, future studies should use an extensive scale for measuring mothers’ irritability rather than only four items. Second, our sample is an Italian sample. Therefore, caution should be used in generalizing results from the present study to other populations; the findings await replication in other cultural contexts. Third, we focused on maternal irritability and harsh parenting, but future research should consider paternal irritability and harsh parenting. Third, we used self-report measure of harsh parenting, which may have led to underreporting rates of the use of these punitive discipline practices due to social desirability. Moreover, because of the Multiple Indicator Cluster Survey’s formulation [26], which asked to mothers whether they or anyone in their household had used each form of harsh parenting against the target child in the last month, we are not completely sure if the mothers refer to themselves or to others when reporting the use of harsh practices. Therefore, even if we are inclined to think that the mothers referred to themselves because there is high correlation (Pearson’s r ≈ 0.60) between this measure and another measure (Discipline Interview, [34]) that explicitly asked mothers to think about themselves in reporting the use of harsh parenting practices, future research should address this limitation. Finally, the reliance only on mother report to assess the level of irritability and harsh parenting could have inflated the estimates of the strength of associations among variables because of shared method variance. For example, because mothers rated both irritability and harsh parenting, the effect of state irritability on harsh parenting may have been biased by the mood (angry versus positive) of the mothers at a given time point. Future studies should adopt a multi-informant approach (i.e., child-report or observation) to corroborate our findings.

Despite these limitations, we maintain that this study represents a step forward in the study of irritability and aggressive behaviors in family contexts. Indeed, to our knowledge there are no previous non-experimental studies that have examined longitudinally the association between maternal irritability and later harsh parenting. Moreover, the findings of the present study have some practical implications in highlighting the need to focus efforts on reducing anger reactions and increasing maternal tolerance for annoying behaviors of children in order to reduce the risk that coercive and aggressive parenting could escalate to harsher forms of discipline and physically abusive parenting.

## Supporting information

S1 Fig Singletrait-multistate (STMS) model of mother’s irritability across four time points (unstandardized solution). (DOCX)

S2 Fig Multitrait-multistate (MTMS) model of mother’s irritability across four time points (unstandardized solution). (DOCX)

S3 Fig LTS model relating mothers’ irritability and harsh parenting. (DOCX)

S1 Data (XLSX)
