# Supplementary material for: Longitudinal relation between state-trait maternal irritability and harsh parenting
Source: PLoS One. 2019 Jan 9;14(1):e0209493. doi: 10.1371/journal.pone.0209493 (PMC6326468; doi:10.1371/journal.pone.0209493)
Supplement: S1 Fig — (DOCX) [file pone.0209493.s001.docx]

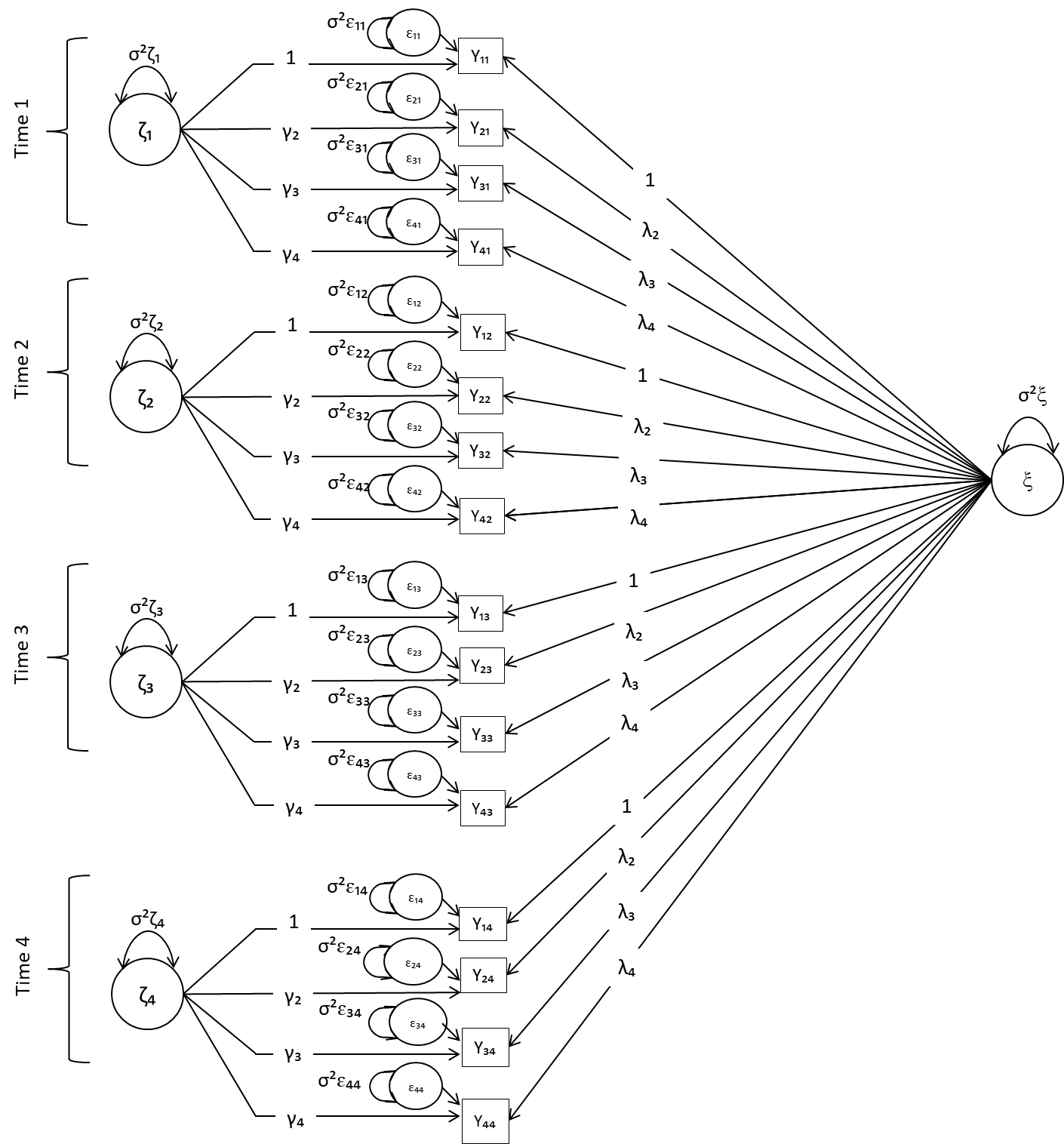


**S1 Fig.** Singletrait-multistate (STMS) model of mother’s irritability across four time points (unstandardized solution)
