# Supplementary figures and images for: Longitudinal relation between state-trait maternal irritability and harsh parenting
Source: PLoS One. 2019 Jan 9;14(1):e0209493. doi: 10.1371/journal.pone.0209493 (PMC6326468; doi:10.1371/journal.pone.0209493)

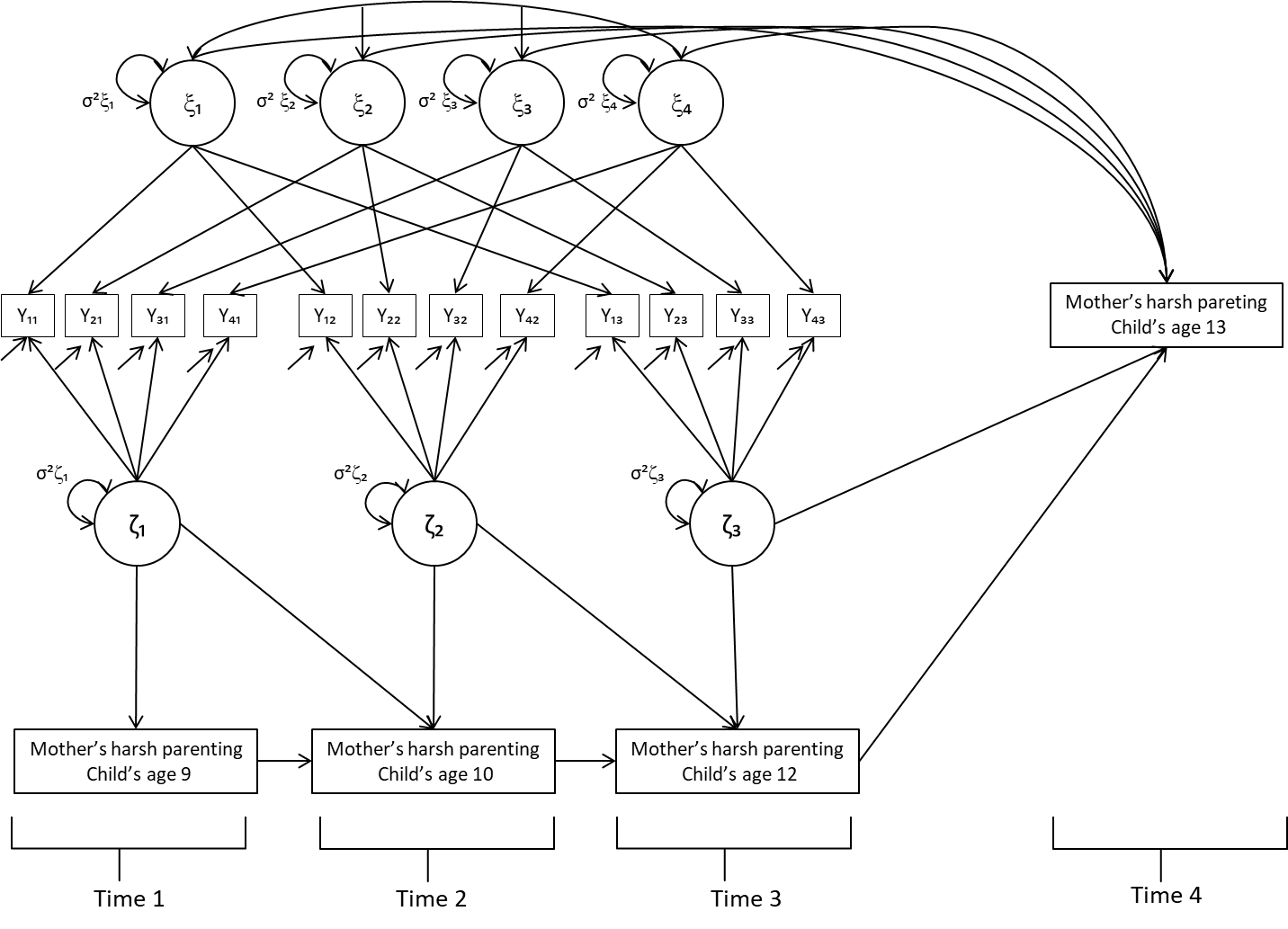


**S3 Fig.** LST model relating mothers’ irritability and harsh parenting.

Supplement: S3 Fig — (DOCX) [file pone.0209493.s003.docx]
